# Genetic structure of the gentle Africanized honey bee population (gAHB) in Puerto Rico

**DOI:** 10.1186/1471-2156-14-65

**Published:** 2013-08-06

**Authors:** Alberto Galindo-Cardona, Jenny P Acevedo-Gonzalez, Bert Rivera-Marchand, Tugrul Giray

**Affiliations:** 1 Department of Crops and AgroEnvironmental Sciences, Agricultural Experimental Station, University of Puerto Rico, Mayaguez, South Botanical Garden, Guayacán St. 1193, San Juan PR 00926, Puerto Rico; 2 Department of Biology, University of Puerto Rico, Natural Sciences, P.O. Box 23360, San Juan 00931, Puerto Rico; 3 Department of Science and Mathematics, Inter American University of Puerto Rico, Bayamon, PR, USA

**Keywords:** *Apis mellifera*, Honeybee population, Hybridization, Africanized, European bees

## Abstract

**Background:**

The Africanized honey bee is one of the most spectacular invasions in the Americas. African bees escaped from apiaries in Brazil in 1956, spread over Americas and by 1994 they were reported in Puerto Rico. In contrast to other places, the oceanic island conditions in Puerto Rico may mean a single introduction and different dynamics of the resident European and new-coming Africanized bees.

To examine the genetic variation of honey bee feral populations and colonies from different locations in Puerto Rico, we used eight known polymorphic microsatellite loci.

**Results:**

In Puerto Rico, gAHB population does not show any genetic structure (*F*_st_ = 0.0783), and is best described as one honey bee population, product of hybridization of AHB and EHB. The genetic variability in this Africanized population was similar to that reported in studies from Texas. We observed that European private allele frequencies are high in all but one locus. This contrasts with mainland Africanized populations, where European allele frequencies are diminished. Two loci with European private alleles, one on Linkage Group 7, known to carry two known defensiveness Quantitative Trait Loci (QTLs), and the other on Linkage Group 1, known to carry three functionally studied genes and 11 candidate genes associated with *Varroa* resistance mechanisms were respectively, significantly greater or lower in European allele frequency than the other loci with European private alleles.

**Conclusions:**

Genetic structure of Puerto Rico gAHB differs from mainland AHB populations, probably representing evolutionary processes on the island.

## Background

Humans and honey bees interacted over history with consequences for distribution of the honey bee, the provider of the first sweetener or honey for humans. Honey bees are naturally distributed throughout Africa, Europe, and the Middle East. In this broad range, they have evolved into 24 subspecies, which have been grouped into three [1], four [2] or five [3] distinct evolutionary branches. Whitfield and colleagues [3] showed using SNP data that honey bees left Africa three times, first to Asia, second to Europe, and third when the African bee *Apis mellifera scutellata* was introduced to America in 1956. Before the introduction of the African honey bees, a subset of European subspecies and other African genotypes were introduced into the New World, first by settlers and later by beekeepers [4-6]. Since their introduction to Brazil in 1956, African bees hybridized with different European honey bees in Americas [5-13]. This was an accidental experiment, where natural hybridization between races of honey bees occurred through time. The result was the Africanized honey bees (AHB), shown to be distinct from the original African race or the European Honey Bees (EHB) in its genetics and behavioral characteristics [14-16]. On the oceanic island of Puerto Rico, AHB have been shown to have a mosaic of traits such as European-like low defensiveness behavior and African-like resistance to ectoparasites e.g. to *Varroa* spp. [14-16]. We dubbed these Puerto Rican bees as gAHB because of their unusual gentle behavior. AHB most likely arrived in Puerto Rico in the first half of 1990s they were reported first in 1994; [17], and now all feral colonies sampled are Africanized (see [14-16], 112 samples, [18], and this study-72 samples).

Hybridization between AHB and EHB has been previously reported in Argentina, Brazil, Perú, and Mexico [5,6,19,20]. The current thinking is that when AHB arrives at a location with a resident European population, there is a large amount of hybridization, followed by elimination of European genes over time, perhaps due to selective advantage of African genes under tropical conditions [3]. The mosaic nature of observed characteristics of gAHB may be better understood with data on population genetics [14-16], potentially providing support for different processes discussed by [16], extending from hybridization to founder effect to selection.

Many studies on population genetics in the Americas have been conducted [5,6,19-22], but in Puerto Rico, the population genetic structure of *A*. *mellifera* is unclear. Natural hybridization between EHB and AHB has been occurring over the last 17 years, and currently, based on mtDNA and morphology, Puerto Rico has a uniform geographic distribution of gAHB, both feral and domestic [14,15]. Previous studies on the genetic structure of honey bees in Puerto Rico relied on the use of morphology, mtDNA markers, and single locus nuclear RFLP markers [14,15]. Other approaches used worldwide to explore the population structure and hybridization zones of honey bees include morphology [13,23] and different molecular genetic markers to study genetic variation i.e. Restriction Fragment Length Polymorphisms and Short Tandem Repeat-microsatellites, SNPs; e.g. [3,24,25]. To further examine the genetic structure of the Puerto Rican honey bee population, we used eight commonly selected microsatellite markers for which historical data on other Africanized and European populations exist [3,21]. These molecular markers could help detect potential source populations for the gAHB and determine any population substructure on the island. One advantage of microsatellites is their high variability that allowed us to use individual-based measures of relatedness to study gene flow and population boundaries. In addition, the microsatellite loci could be spread across the genome, and occur at identifiable specific locations [26]. Here we chose only eight microsatellite loci shown to be highly informative [21,26]. More loci could be added since there are many microsatellite loci developed and published for *A*. *mellifera* (e.g. 250 reported in [26]).

The study of genetic structure and variation of gAHB will allow us to test two alternate hypotheses to explain the mix of AHB and EHB traits in this population: Structure and Hybridization hypotheses. Testing of these hypotheses provides a first approach to detect isolation local and global selection:

The “*Structure hypothesis*” is presence of different composition of European or African alleles in different subpopulations across the island of Puerto Rico. This could be due to local selection or isolation due to geographic barriers (Puerto Rico is composed of one main island and two adjacent islands and exhibit six life zones with different climate and floral phenology). In the local selection scenario, alternate European or African traits that best respond to environmental pressure under different conditions would be selected in different regions across Puerto Rico. According to this hypothesis different levels of European genes could be maintained in populations of feral bees at geographically distinct parts of the island, due to the different environmental conditions. Puerto Rico has a topography that has a strong effect on the climate, with the wetter regions on the windward, northern side of the mountains, and drier climate in the leeward rain shadow [27]. Moreover, Puerto Rico’s six different life zones [28,29], could provide opportunity for ecologically adapted, isolated population subdivisions. We could therefore expect European traits, for instance, on higher cooler regions of the island. In the isolation scenario, populations with high European genes in isolated regions such as surrounding islands may be observed. Structure hypothesis then predicts genetic differences among delimited geographic areas, in other words, a genetic structure for honey bee subpopulations in Puerto Rico. This will also predict a departure from Hardy-Weinberg Equilibrium (HWE) for different marker loci, indicating population substructure due to genetic drift or due to local selection on associated loci.

Alternately, the “*Hybridization hypothesis*” based on previous studies, states that there is only one gAHB population with large introgression of European genes, as demonstrated in one nuclear locus [14-16]. According to the hybridization hypothesis, there is no clear differentiation of subpopulations of gAHB in Puerto Rico. This could be due to a brief and effective hybridization of AHB and EHB, with a restricted flow of African genes from outside, followed by a spread of the hybrid bee across the small island. Any selective advantage for alternate AHB or EHB alleles would be present early, and spread to the whole population under a global selection scenario. In absence of a selective advantage for AHB or EHB alleles, hybridization hypothesis predicts that there will be a single, unstructured bee population with similar introgression of European genes across the sampled molecular markers. In the absence of additional Africanized bees breeding and spreading into the newly occupied territory, the sampled African alleles in Puerto Rico should not be increasing over time, but remain similar to the initial hybrid population. Alternately, if certain AHB or EHB alleles were to confer an early and fast selective advantage, hybridization hypothesis predicts that there will be different levels of introgression of EHB genes, commensurate with selective advantage at different loci, but with no current deviation from HWE. Although few known markers would be adequate to test the immediate prediction of a uniform genetic structure as per the hybridization hypothesis, early and fast hybridization would make it very unlikely to detect global selection at a resolution of 8 microsatellite markers. Future greater density marker studies, such as 1000 s of SNPs identified in SNP arrays [3,30] or RAD-TAG sequencing with potential to generate multiples of 10000 SNPs [31] is more likely to provide resolution needed to determine regions of genome responding to alternate global selection pressures.

## Methods

### Sample collection and DNA extraction

**Figure 1:**
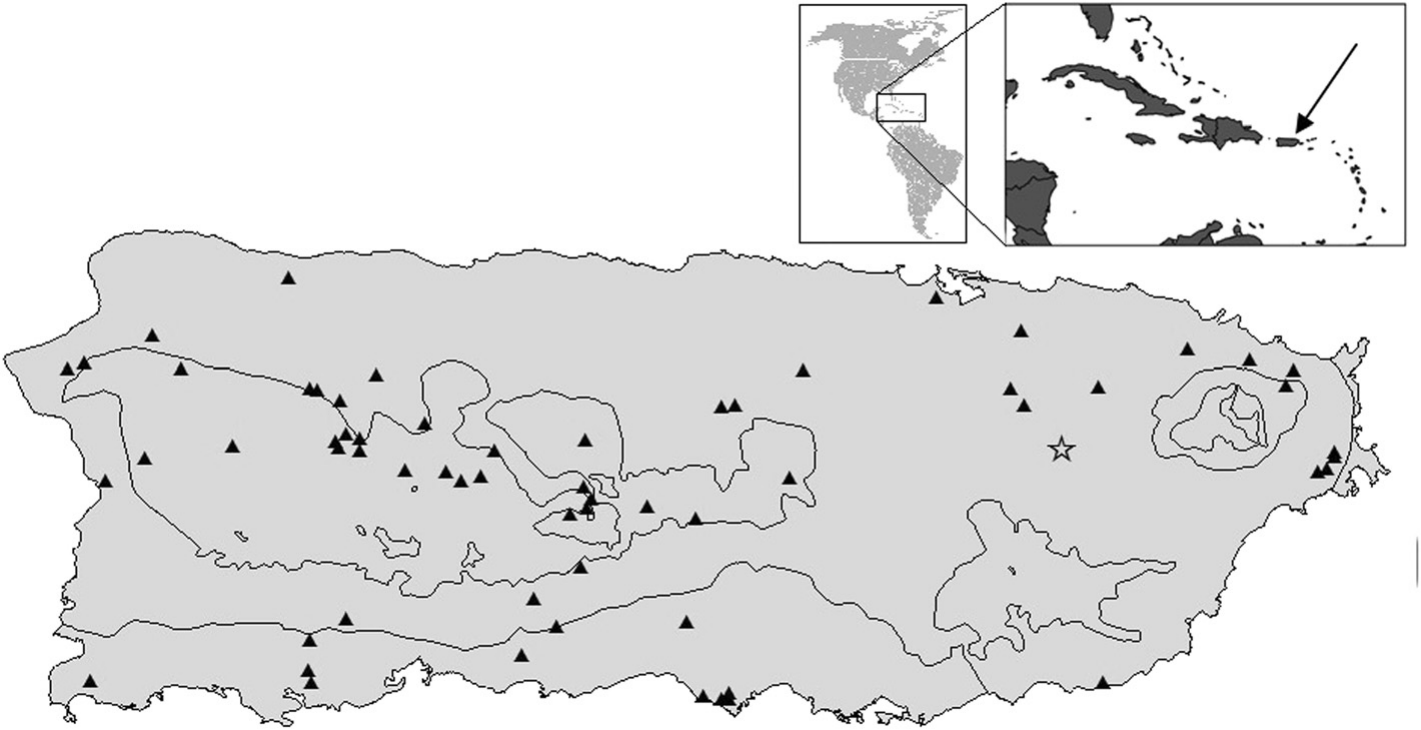
Map of Puerto Rico with apiaries sampled (triangle) and the research apiary (star).

The main island of Puerto Rico lies between 17°45′ N and 18°30′ N, and its longitude ranges from about 65°45′ W to 67°15′ W (Figure 1). With 8740 km^2^ surface area, Puerto Rico is the smallest and eastern most of the Greater Antilles. Vieques and Culebra Islands lie a short distance, 10 km southeast and 27 km east of the main island, respectively. Vieques and Culebra are the westernmost of the Lesser Antilles, which extend in a southeasterly arc from Puerto Rico to the northern coast of South America [32].

A total of 72 colony samples were collected from several apiaries and feral colonies from different places in Puerto Rico: Main island (55), Vieques (17). This is a large sample for population genetic studies using microsatellite markers with high levels of polymorphism [33]. We also included 11samples from the typical European bee commercially available to Puerto Rico, the Italian bee or *Apis mellifera ligustica*, purchased from Hawaii (11). We include more samples from the Vieques island because it is separated from Puerto Rico by a mass of water and may provide an important test for the *Structure hypothesis*. In addition, in 1988 a program of producing European queen bees was established in Vieques. This discontinued program was established because since that date, Puerto Rico was quarantined against import of European bees from elsewhere (except Hawaii) to prevent introduction of diseases and parasites of the honey bee [34]. However, in our studies we have not encountered EHB morphology or mtDNA on repeated sampling from Vieques [14-16] and this study.

Briefly, we used the protocol from [24] to identify mtDNA from Africanized (AHB) and Eastern European (*A*.*m*. *ligustica* or Italian or EHB) bees, as described by [14]. Previous studies had demonstrated for Puerto Rico only maternal descent of these two types [16]. We amplified a region of the rDNA of 964 bp. The products from PCR were later subjected to digestion with the restriction enzyme EcoR1. This enzyme digests only the EHB product to two fragments of nearly equal size (484 and 480 bp), resulting in a band that moves further on the gel in comparison to AHB band. The bands were visualized after running in a 1% agarose gel and staining with ethidium bromide.

Bees were stored in 95% ethanol and kept at 4°C until processed. To prevent oversampling of maternal alleles, only one bee per colony was subjected to genetic analysis. Genomic DNA from an individual honey bee thorax was extracted using DNeasy extraction kit from QIAGEN® with the animal tissue protocol.

### Microsatellite analysis

We used eight (A14, A35, A79, A88, A107, A113, ED1 and IM) DNA microsatellite loci [35-37] that were used to study other AHB populations e.g. [21]. These markers were selected to compare our results to the published results on AHB in the New World [21,22]. To score the microsatellite loci, we used a modified inexpensive protocol for fluorescent tagging including the addition of a M13 sequence tail (CACGACGTTGTAAAACGAC). This M13-tail protocol consists of using 3 primers during the PCR: a sequence-specific forward primer with M13 tail at the 5′ end, a sequence-specific reverse primer and the universal fluorescent-labeled M13 tail. Four different M13 tails were used, each labeled with a different fluorescent dye (FAM, VIC, PET, NED) [38]. The sequences of the original primers used, excluding the tail, are given in Table 1. The raw data were revised and adjusted by eye and determined the allele size nearest to the base, e.g. if the fragment values were 170.2, the size was 170, and if it was 170.8, nearest to the base size was 171. When compared with data obtained by [21], for the products amplified with M13 tail were found 19 nucleotides longer. The 19 bases were removed to obtain fragment sizes comparable to obtained for Texas. Different alleles were inferred only with size differences matching multiples of repeat size for the locus across Puerto Rico and Texas.

**Table 1:** List of the primers sequences of microsatellite loci and the fluorescent dyes

**Locus**	**Primer sequences**	**Fluorescent dye**
A107^α^	F: M13-CCGTGGGAGGTTTATTGTCG	NED
	R: CCTTCGTAACGGATGACACC	
A113^α^	F: M13-CTCGAATCGTGGCGTCC	PET
	R: CCTGTATTTTGCAACCTCGC	
A79^α^	F: M13-CGAAGGTTGCGGAGTCCTC	NED
	R: GTCGTCGGACCGATGCG	
A14^α^	F: M13-GTGTCGCAATCGACGTAACC	VIC
	R: GTCGAlTACCGATCGTGACG	
A35^β^	F: M13-GTACACGGTTGCACGGITTG	FAM
	R: CTTCGATGGTCGITTGTACCC	
A88^β^	F: M13-CGAATTAACCGATTTGTCG	PET
	R: GATCGCAATTATTGAAGGAG	
ED1^γ^	F: M13-CAACAGCCGTGAACGCTATC	NED
	R: TCATCGTGTACCAATAACG	
IM^γ^	F: M13-ACGCAAATGACAAGTATTAG	PET
	R: GAGTGTATTTCGAAATCGATG	

^α^ Estoup et al. 1994.

^β^ Estoup et al. 1995.

^γ^ Rowe et al. 1997.

We optimized PCR conditions in a gradient thermo cycler (Biorad-MyCycler). Final 8 μl reactions were performed with following conditions: 10 ng/μL of genomic DNA, 2.5 units of QIAGEN Taq Master Mix, 0.30 μM of the unmodified primer, 0.02 μM of the primer with the M13 tail and 0.30 μM of the fluorescently labeled M13 primer (6-FAM, NED, VIC or PET; Applied Biosystems®). After a first 5-min denaturing step (94°C), our PCR protocol consisted of 30 cycles of: 30 s at 94°C, 30 s at the primer-specific annealing temperature (50 to 60°C) and 1 min at 72°C followed by a final extension step of 20 min at 72°C. Genotyping was run on an automated station ABI 3130xl Genetic Analyzer (Applied Biosystems®) using LIZ-500 size standard and allele sizes were scored using Gene mapper version 4.0 (Applied Biosystems®). On occasion a locus did not amplify in individual samples (see Table 2), however, in general all loci were present in every population. For allele calls, two replicates for each individual were run to confirm the allele was not a PCR artifact. Also reviewers examined the data generated in GeneMapper to confirm the observed allele size. All revisions were done by visual discrimination.

**Table 2:** **Loci, sample size (N), no. alleles (Na), no. effective alleles (Ne), no. alleles private to European (NaPE) bees in previous studies, observed heterozygosity (Ho), expected (He) and unbiased expected heterozygosity (UHe), average departure of genotype frequencies within populations (*F***_**is**_**), correlation between gametes within an individual relative to the entire population (*F***_**it**_**), and fixation index (*F***_**st**_**) by population for codominant data Puerto Rico (PR) and Vieques (VIE)**

**Pop**	**Locus**	**N**	**Na**	**Ne**	**Ho**	**He**	**UHe**	**F**
**PR**	**107**	50	13	6.211	0.760	0.839	0.847	0.094
	**113**	54	9	2.839	0.611	0.648	0.654	0.057
	**79**	53	11	5.989	0.868	0.833	0.841	−0.042
	**A14**	53	9	5.579	0.830	0.821	0.829	−0.011
	**A35**	52	9	3.930	0.596	0.746	0.753	0.200
	**A88**	54	5	2.827	0.722	0.646	0.652	−0.118
	**ED**	47	12	5.102	0.723	0.804	0.813	0.100
	**IM**	51	8	4.401	0.745	0.773	0.780	0.036
**VIE**	**107**	15	6	3.913	0.933	0.744	0.770	−0.254
	**113**	17	6	2.524	0.647	0.604	0.622	−0.072
	**79**	16	7	3.631	0.563	0.725	0.748	0.224
	**A14**	16	9	6.169	1.000	0.838	0.865	−0.193
	**A35**	17	8	3.229	0.588	0.690	0.711	0.148
	**A88**	16	6	3.765	0.563	0.734	0.758	0.234
	**ED**	16	8	4.697	0.813	0.787	0.813	−0.032
	**IM**	15	6	5.422	0.800	0.816	0.844	0.019
**All Pops.**	**Locus**	**Ht**	**Mean He**	**Mean Ho**	**NaPE**	***F***_**is**_	***F***_**it**_	***F***_**st**_
	**107**	0.807	0.792	0.847	6*	−0.069	−0.049	0.019
	**113**	0.635	0.626	0.629	1*	−0.005	0.010	0.015
	**79**	0.791	0.779	0.715	1*	0.082	0.095	0.015
	**A14**	0.836	0.829	0.915	1*	−0.103	−0.095	0.008
	**A35**	0.725	0.718	0.592	1*	0.175	0.183	0.009
	**A88**	0.696	0.690	0.642	2**	0.069	0.077	0.008
	**ED**	0.827	0.796	0.768	0	0.035	0.071	0.038
	**IM**	0.814	0.794	0.773	0	0.027	0.051	0.024

*Shared with Pinto et al. (2005).

**Shared with Kraus et al. (2007).

### Statistical analysis

The number of alleles, allele frequencies, and observed (Ho) and expected (He) heterozygosity of each microsatellite locus were estimated using GENEPOP 4.0 [39]. GENEPOP 4.0 was also used to test for linkage disequilibrium and HWE. A regression analysis was used to relate the allelic frequency of European population from Puerto Rico with the reference European population. The reference European population was taken from [21,22]. Our results were compared with Texas results from [21] that were divided in two groups: first (1991-1996) and second (1997-2001) group, representing early and later stages of Africanization. Estimates of population structure and gene flow were assessed using F-statistics, also with GENEPOP 4.0. Unbiased gene diversity (*Hd*), and allelic richness (*Rs*) per locus and population sample were computed using FSTAT version 2.9.3 package [40]. In FSTAT, the sample size for allelic richness estimation is fixed as the smallest number of individuals typed for a locus in a sample, which in this study was 72 (Locus ED1). Population structure was also analyzed using a Bayesian model-based clustering method provided in STRUCTURE 2.3.3 [41,42]. This program infers structure of populations using allele frequencies of unlinked markers (microsatellites). The parameter set was programmed for independent alleles and individuals to have a mixed ancestry. The program was asked to place individuals from all populations into one and two groups, and in three runs the analyses consisted of 10000, 100000, and 1000000 burn-in replicates, and run lengths of 10000, 100000, and 1000000 replicates. The best estimate of K or the number of populations was determined by looking at the values of log Pr(X/K) and the value of α [41,42]. In structure analysis we did not use Culebra samples, because the low sample number dissuaded us from doing the comparison. Single and multiloci Fst values were estimated using the weighed analysis of variance method by [43] to measure genetic differentiation with the GENETIX 4.02 program [44]. We analyzed distant locations that appeared to be genealogically close on an unrooted neighbor joining tree based on genetic distance, constructed using the Phylip 3.57 program [45].

## Results

### Genetic diversity

The number of alleles per locus varied between six (loci A88) and 19 (loci A79) (Table 2, and Additional file 1: Table S1). Gene diversity varied between 0.611 and 0.881. The loci A107, A79 and A35 were more polymorphic and the locus A113 showed low heterozygosity values (Table 2). Our data indicated a mean number of 12.75 alleles, a mean number of 7.4 new Africanized and European alleles in comparison to [21,22], and a mean of 5.4 alleles per locus for the eight loci shared with Texas and Mexico (Table 3). Mean allelic richness ranged from 5.9 (locus A88) to 15.0 (locus A79), and lastly, the mean gene diversity ranged from 0.61 (locus A113) to 0.88 (locus A88), across all population samples (Table 3). Paired *t*-test of honey bees from Puerto Rico and Texas showed bees did not differ in genetic diversity (*Hd*) with the population of first group or early Africanization period (TX 91-96) (t = 0.67, df = 7, *P* = 0.51), and had a difference with the second group or late Africanization period (TX 97-01) (t = -2.03, df = 7, *P* = 0.06) (Table 3). The test on allelic richness (*Rs*) shows the same pattern, with gAHB showing no significant differences from the first group (t = 2.37, df = 7, *P* = 0.11) and significant differences from the second group (t = 1.09, df = 7, *P* = 0.05).

**Table 3:** Loci, number of alleles per locus (Na), unbiased gene diversity (Hd), allelic richness (Rs) to Texas (TX1991/1996, TX1997/2001, *Pinto 2003) and new alleles to Puerto Rico (PR2007)

TX1991/1996*				TX1997/2001*			**PR2007**			
**Loci**	**Na**	**Hd**	**Rs**	**Na**	**Hd**	**Rs**	**Na**	**Hd**	**Rs**	**New alleles**
**A107**	30	0.938	12.083	27	0.944	12.748	13	0.825	14.923	9
**A113**	15	0.743	6.451	13	0.828	7.488	9	0.611	6.992	3
**A79**	16	0.794	7.096	17	0.910	10.035	11	0.881	14.992	11
**A14**	23	0.845	8.966	26	0.910	11.077	10	0.851	9.925	2
**A35**	19	0.690	7.102	19	0.902	9.926	11	0.834	14.625	11
**A88**	15	0.721	5.285	16	0.844	8.181	6	0.683	5.944	2
**ED1**	14	0.526	5.211	17	0.796	7.728	13	0.850	12.000	12
**IM**	10	0.796	5.759	11	0.833	7.144	8	0.814	7.962	9

### Population genetic structure

**Figure 2:**
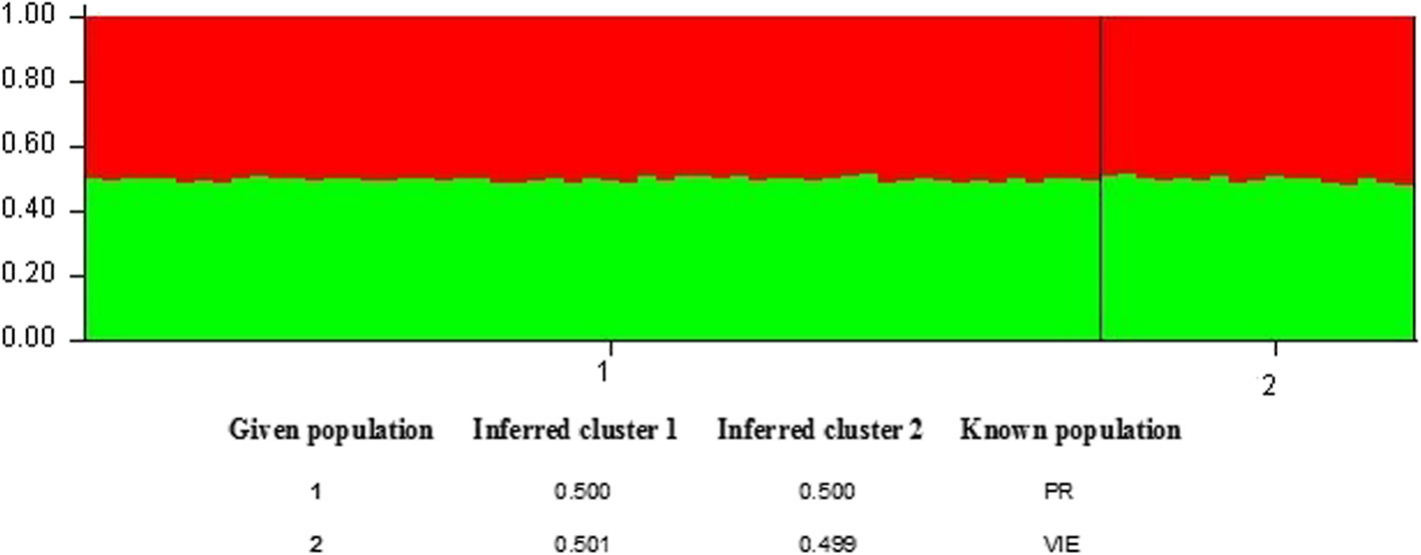
**Bar plot produced by STRUCTURE assuming 2 populations showed no separation.** 1 and 2 indicate the samples from the island of Pueto Rico (1) and the satellite island, Vieques (2). And proportion of membership of each pre-defined population in 2 clusters.

**Figure 3:**
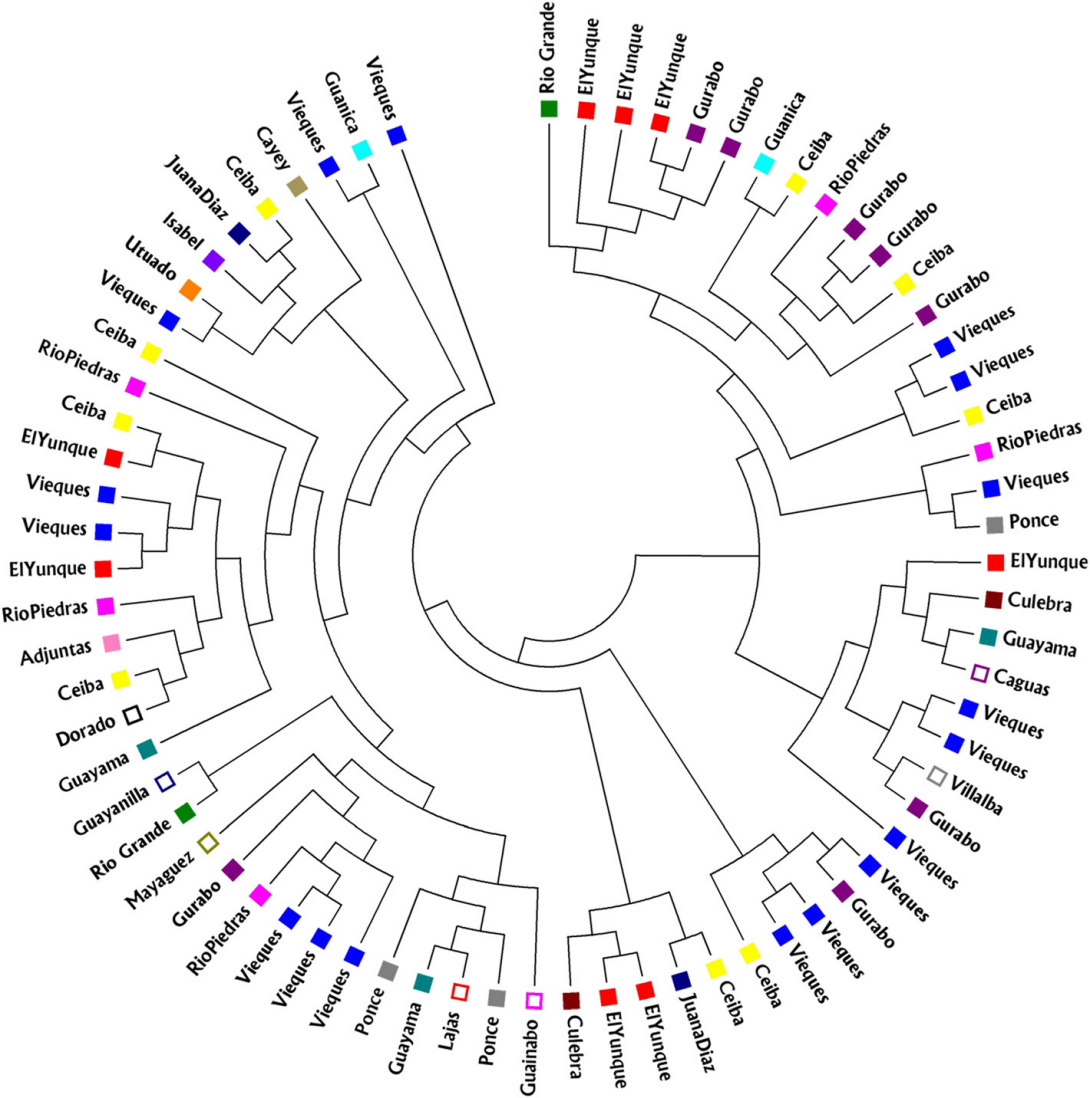
Neighbor-joining tree, showing the genetic relationships among each individual member of sample locations in Puerto Rico, inferred from microsatellite data.

The software program STRUCTURE did not separate sampled gAHB individuals into different populations. In Puerto Rico, gAHB population does not show any genetic structure (*F*_st_ = 0.0783) (Table 2), and is best described as one honey bee population (Figure 2), product of hybridization of AHB and EHB. All the loci showed similar low genetic differentiation in the Puerto Rico honey bee population (Table 2). In fact, the phylogenetic analyses revealed all sampled provinces, including those separated by geographical barriers to cluster together, regardless of location (Figure 3).

In Puerto Rico, we found that 18 percent of European alleles are shared with the Texas population, in eight microsatellite loci. Exact tests for linkage disequilibrium at 5% level for each pair of loci did not provide any significant P-value after Bonferroni corrections.

For these eight markers, we identified an average of 12.75 alleles by loci with heterozygosities in concordance with the expectation based on the HWE. We identified 12 (11.8 %) European private alleles in six of the eight microsatellite loci sampled to be present in the gAHB population (Table 3). The presence of all previously reported European private alleles in the gAHB population suggests that individuals have a mixed genetic origin. The locus A107 has six of these private European alleles, and loci A113, A79, A14 and A35 have one private European allele each. The locus A88 has two alleles exclusively European [22], and that were not present in the population studied by Pinto and colleagues [21]. In Puerto Rico, these two private European alleles are present at a similar frequency to that reported in Hawaii EHB European population [22].

**Figure 4:**
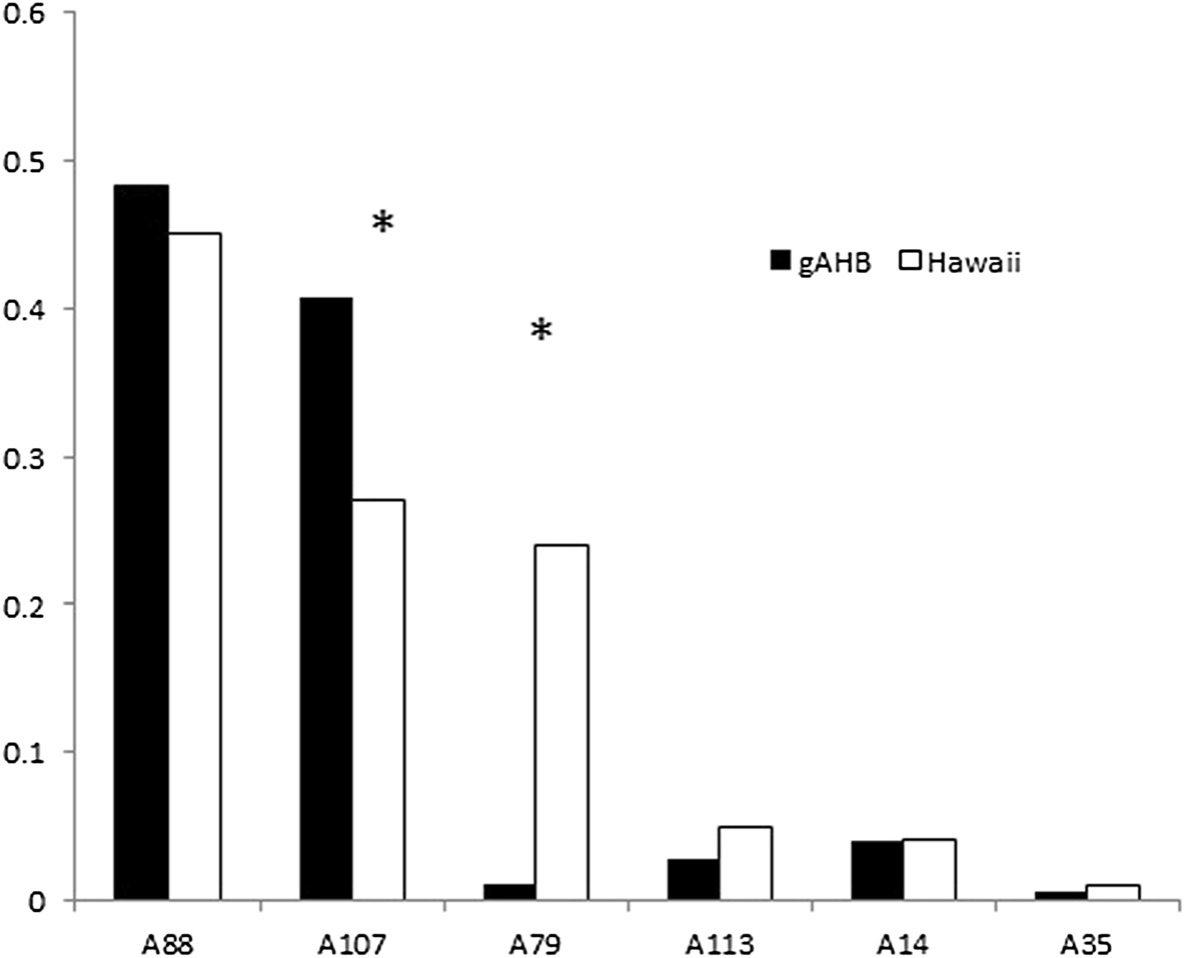
**Comparison of the European allele frequency (E) among six microsatellite loci in a Hawaiian (Hawaii) and Puerto Rican (gAHB) bee population.** The star (*) indicates the two loci where the difference was significant, A107 (*X*^2^ = 6.60, df = 1, P < < 0.0001) and A79 (*X*^2^ = 39.12, df = 1, P < 0.01).

There is a significant correlation between the frequencies of European private alleles in Hawaii population and in gAHB population for the six loci studied [21,22] (R^2^ = 0.71, *P* = 0.03). We found that in Puerto Rico, four loci (A113, A14, A35 and A88) show similar allelic frequencies as the Hawaii EHB population. In contrast, the locus A107 that represents 50 percent of private alleles, has a higher allelic frequency for these European alleles even than in the EHB reference population (*X*^2^ = 6.60, df = 1, *P* < 0.01) (Figure 4). The locus A79 has a significantly lower European allelic frequency than expected (*X*^2^ = 39.12, df = 1, *P* < 0.01) (Figure 4).

We found a few alleles only in Vieques that were not found in Puerto Rico, but their frequency was low. Large numbers of markers studied in other AHB populations are needed to determine the African ancestry of Puerto Rican gAHB population. Nevertheless, a genetic distance analysis revealed gAHB to be closer to Texas population than to the Brazil population (results not shown), indicating a more northern source for AHB in Puerto Rico than Brazil, and consistent with speculated Texas origin [16].

## Discussion

The population genetics analysis indicates the presence of a uniform genetic structure or a single gAHB population in Puerto Rico, consistent with the hybridization hypothesis. Interestingly, two marker loci show introgression levels for EHB alleles significantly different than for other loci tested. This is in agreement with a global selection scenario for AHB or EHB alleles for different traits.

One issue with highly polymorphic marker loci has been the perceived need to obtain a large sample [33]. This probably is important especially if the “population” is actually composed of multiple subdivisions. Because we found a single population in Puerto Rico (including Vieques), the sample size of 72 is well above the recommended 20 to 25 for a single population, in relevant reference for the recent analysis. In addition, we ran multiple tests, based on allele frequencies, and also based on individual genotypes, giving the same result of a single population without subdivisions (see results and Figure 3). In a further attempt to test the accuracy of these analyses, we also performed the individual assignment to the Vieques and main island “populations” using GeneClass2 [46] and also a spatial PCA using Adegenet on R platform. All four analyses gave similar results (analyses not shown), confirming the inference of a single continuous population on Puerto Rico.

The continuous uniform population could be the result of the large range of movement of the bees in comparison to the size of the island. For instance, reproductive drone congregation areas, DCAs are found at about 1.5 miles radius around bee colonies, and less than 10 such DCAs can span the island from North to South [47]. Two other factors that may contribute to the homogenization of bee population in Puerto Rico are the formation of swarms and adoption of feral swarms by beekeepers. A swarm is a colony division, which involves migration of a part of the individuals. This movement of feral colonies occurs throughout the year, but their frequency is high at the beginning of the mating season (May to October, [17]). Second, an activity that is common for beekeepers around the island is to adopt feral bee colonies to populate their commercial hives. Regularly replacing queens of commercial colonies is considered a good beekeeping practice. However, in Puerto Rico only few beekeepers introduce new queens to their colonies. These queens are mainly from Hawaii (EHB), and rarely from other places in the United States. These factors would contribute to observed genotypic homogenization throughout Puerto Rico.

The variability of the microsatellite loci in the honey bee population in Puerto Rico is similar to that found in the EHB populations for a detected number of alleles and the values of gene diversity [21], (Table 3). Pinto and colleagues [21], found that their results were divided in two temporal groups, first (1991-1996) and second (1997-2001). These groups illustrate a pattern of genotypic differentiation, which shows that the first group was more similar to EHB and the second group more similar to AHB. When these two groups were compared with Puerto Rico, we found that Puerto Rican honey bee populations are more similar to the first group (European-like), lacking the temporal component indicating change towards more Africanized features as reported in Texas [21]. In Puerto Rico, we used a paired *t*-test for eight loci used both in this study, and reported previously [21] for heterozygosity comparison. We found that the stable Africanized population in Texas (years 1997-2001) had higher heterozygosity, than the Puerto Rico population even when the allelic richness was comparable (positive yet non-significant t). These results may mean that after the original introduction of AHB, there has been no or little influx of new AHB to Puerto Rico, probably because of its island nature. The increased Africanized genes and heterozygosity in AHB population in the mainland (i.e. Texas) could be due to the many spatial and temporal points of entry available to the AHB and selection for Africanized traits in the mainland. In Puerto Rico, it is safe to assume that these arrivals of new AHB are very low. This restricted flow of AHB to the island could explain the genetic difference observed with the late Texas population (Table 3). The early impoverished EHB genes due to loss of feral bees to *Varroa* mites both in the mainland and in Puerto Rico, could contribute to the similarity [16]. The Puerto Rican Africanized bees would be more similar to early AHB population in Texas in absence of constant AHB gene flow. However, under hybridization without selection, all markers across the genome should show similar introgression levels. Only hybridization followed by alternate alleles selected for different loci could explain the pattern observed in this study.

Introgression is a genetic invasion of a local genome by a foreign genome [48-52]. Although one idea would be that reduced defensiveness is the result of interbreeding throughout the island [14-16], it is intriguing to find that the population maintains Africanized features such as small size, high parasite defenses, and Africanized mtDNA [16]. Therefore selection on particular traits, in particular regions of the genome globally across the island population is the favored hypothesis. In fact, there are two exceptional loci that may indicate presence of selection for European or African genes near the loci. First, there may have been selection for European genes near the locus A107. The locus A107 showed a higher frequency of European alleles in Puerto Rico, when compared with pure EHB populations (Figure 4). Moreover, this locus has 50 percent of all the European private alleles we found in this study. This A107 locus is on the same linkage group (LG7) that also contains the two Quantitative Trait Loci for aggressive behavior (Sting 1 and 2 [53]).

**Figure 5:**
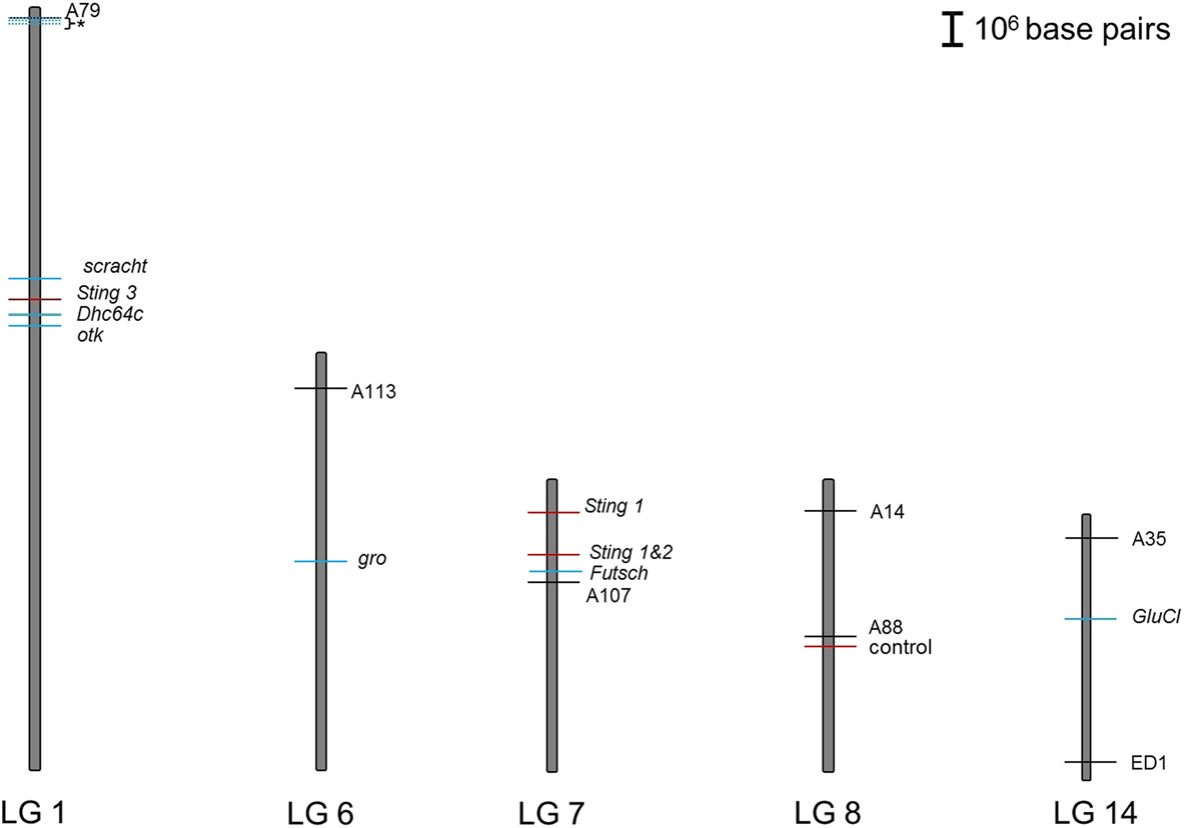
**Microsatellite loci A79, A113, A107, A14, A88, A35 and ED1 (black lines) associated to linkage groups with aggressive behavior Quantitative Trait Loci Sting 1, 2, and 3 (red line) [**[51]**] and *Varroa *resistance genes (blue lines): scracht (scrt), Dynein heavy chain 64C (Dhc64c), Immunoglobulin gene Superfamily (otk), groucho (gro), Futsch, paralytic (para), fringe (fng), Glutamate-gated Cl- channel (GluCl) [**[52]**] more three groups that including 11 new gene to *Varroa Sensitive Hygiene Behavior *[**[57]**].**

In contrast, another locus, A79, that is on the same Linkage Group as *Varroa* mite resistance related genes (identified in an expression study [54]), showed significantly greater Africanized allele frequency (Figure 5). This same linkage group also carries the QTL Sting 3, and this may explain variation in defensive response of gAHB colonies described elsewhere [14-16]. *Varroa* has been an important pest on the island, and resulted in diminished bee populations, as can be assessed from honey bee collection data reported by [16]. Currently, the virulent Korean haplotype of *Varroa destructor* is present on the island yet does not lead to colony losses or overt symptoms such as viral damage to bees (unpublished results, Jenny Acevedo, Alberto Galindo, and Tugrul Giray, see also [55]).

The deviations in allele frequencies for specific loci may be tentative evidence for global selection for a mosaic of African-like and European-like alleles. This strong global selection may have followed soon after hybridization process since the markers and candidate genes are relatively distant considering the high rate of recombination observed in honey bees [56]. If this is true, for gAHB we predict that LG7 will be mostly European, associated with lower aggressive behavior, and LG1 would be mostly African, associated with greater *Varroa* mite defenses. This hypothesis of fast evolution or selection was suggested previously [16]. An alternate hypothesis that could address the linkage on the face of high recombination rate is presence of other linked genes associated with the trait, especially for *Varroa* resistance on LG1, near the A79 marker. Interestingly, in the time this paper was under review, a new publication identified 11 more candidate genes on LG1 in a linkage study for Varroa resistance [57], supporting the prediction of the *more linked genes* hypothesis (Figure 5). The two hypotheses, fast evolution and more linked genes, are not mutually exclusive, in fact they could be synergistic in explaining the markers associated with trait genes even with high rate of recombination. Both hypotheses can be tested in future studies by use of high density molecular markers to be produced by next-generation sequencing methods.

The long-term stability of distribution of EHB-AHB allele frequencies suggests a stable equilibrium with reduced effects of genetic drift [58]. The high genetic similarity of honey bee populations in Puerto Rico to initial phase of AHB Texas population, with large EHB contribution is interesting considering the AHB has now been in Puerto Rico for over 18 years. This again suggests an environment different than the mainland subtropical conditions [15,16]. Because behavioral and now population genetics data indicate formation of a distinct gAHB population in Puerto Rico, the limited entry of European queens to the island, and potential rapid evolution and mosaic selection scenarios [16], make it important to examine origin and evolution of gAHB using high density molecular markers.

## Conclusions

We observed that European private allele frequencies are high in all but one locus. This contrasts with mainland Africanized populations, where European allele frequencies are greatly diminished. Two loci with European private alleles, one linked to two known defensiveness QTLs, and the other to 11 known genes associated with *Varroa* resistance mechanisms were respectively, significantly greater or lower in European allele frequency than the other loci with European private alleles. Taken together, this evidence supports retention of a mosaic of African and European alleles for certain genes, mirroring the mosaic of African and European traits observed in bees from this gAHB population.

We consider this work is important for population geneticists and evolutionary biologists, because Africanization of honey bee in Puerto Rico is relatively recent, and combination of alleles from European and African bees presented in this study has implications for evolutionary processes on islands. Sociogenomics researchers may be interested in this population with increasing use of functional genomics and next-gen sequencing to study mechanisms underlying described variation in behavior of honey bees.

## Competing interests

The authors declare that they have no competing interests.

## Authors’ contributions

AGC generated the hypothesis and AGC and TG generated the study design. Sample collection was conducted by AGC and BRM. TG served as primary graduate mentor for AGC. Genotyping was completed by AGC and JPA. Statistical analysis was conducted by AGC in collaboration with TG. All authors were involved in scientific discussion of the project and wrote the paper. All authors read and approved the final manuscript.

## Supplementary Material

Additional file 1: Table S1 “Allele frequency data for eight microsatellite loci used to compare PR and VIE populations”. Click here for file
